# A bibliometric and systematic review of the Methods for the Improvement of Vulnerability Assessment in Europe framework: A guide for the development of further multi-hazard holistic framework

**DOI:** 10.4102/jamba.v15i1.1486

**Published:** 2023-12-27

**Authors:** Ali Jamshed, Irfan A. Rana, Joern Birkmann, Joanna M. McMillan, Stefan Kienberger

**Affiliations:** 1 Institute of Spatial and Regional Planning, Faculty of Civil and Environmental Planning, University of Stuttgart, Stuttgart, Germany; 2 Department of Urban and Regional Planning, National University of Sciences and Technology (NUST), Islamabad, Pakistan; 3 Department of Geoinformatics – Z_GIS, University of Salzbur, Salzburg, Austria

**Keywords:** vulnerability, disaster risk, climate change adaptation, frameworks, IPCC, bibliometric analysis

## Abstract

Conceptual frameworks are vital for identifying relevant components, dimensions and indicators to assess vulnerability to natural hazards and climatic change. Given the fact that vulnerability is applied and used in various disciplines and by multiple schools of thought, several conceptual frameworks to assess and conceptualise vulnerability have been developed. Even though these frameworks have been widely cited in research, the range and context of application and contextual use of such frameworks have rarely been explored. This paper provides a systematic review of the MOVE (Methods for the Improvement of Vulnerability Assessment in Europe) framework. Bibliometric and systematic analyses were performed to better understand who and how the MOVE framework has been taken up by other researchers. The MOVE framework has been widely cited in different research fields. Several studies directly used the framework for assessing vulnerability both in terms of its factors and the different thematic dimensions of vulnerability (e.g. social, physical, ecological). Some studies have used it as a basis for developing context-specific studies of vulnerability and risk assessment frameworks. Finally, we also discuss critiques of the MOVE framework that can provide direction for future vulnerability assessments.

**Contribution:**

Critique of the MOVE framework can be helpful in further improvement and development of a multi-hazard holistic framework that would be flexible enough to support multiple theoretical perspectives in disaster risk and climate change discourses.

## Introduction

Vulnerability mean the fragility of living and non-living things (Luna 2018). The concept has gained significant attention globally in recent decades and has been used in various scientific discourses, for example, human ecology, human geography, development, poverty studies, disaster risk reduction (DRR) and climate change adaptation research (Birkmann 2013; Jamshed 2021). Multiple schools of thought have adapted the concept for their purposes and according to their norms, and therefore, the concept has various definitions and interpretations (Adger 2006; Birkmann 2013; Birkmann & McMillan 2020). In disaster risk science, vulnerability was once viewed in terms of physical structures and disaster considered identical to the external hazard. Accordingly, vulnerability was just a matter of exposure to a hazard. Later vulnerability was recognised as a property of social and ecological systems (Cardona et al. 2012). The concept has become crucial in disaster and climate change science and is used extensively.

Initially, vulnerability was considered as ‘the potential of loss’ (e.g., Mitchell 1989) or being exposed and affected (e.g., Cutter 1993) by hazards while focussing on individuals. Later, the focus shifted to social and economic characteristics of individuals and groups (e.g., Adger 1999; Blaikie et al. 1994) that supported the capacity to cope with hazards or climate change. According to this view, vulnerability was the absence or diminishment of capacity to cope. Subsequently, vulnerability was seen from a system-oriented perspective, that is, characteristics of different systems (e.g., human, physical, environmental systems) that allow human and ecological systems to be adversely affected by a hazard (Birkmann et al. 2013; Intergovernmental Panel on Climate Change [IPCC] 2014; Turner et al. 2003).

The advancement in the concept of vulnerability and different understandings in various schools of thought led to different frameworks for assessing vulnerability. Bohle (2001) referred to the ‘double structure of vulnerability’ and Wisner et al. (2004) proposed a ‘pressure and release’ view of vulnerability that incorporated a political economy perspective. Turner et al. (2003) put the socio-ecological perspective at the centre of vulnerability analysis. A holistic perspective is presented by Cardona and Barbat (2000) in their ‘holistic framework for vulnerability and risk assessment’; Birkmann (2006) provided the ‘BBC framework’ that was a precursor of the MOVE framework (Birkmann 2013). Füssel and Klein (2006) and IPCC (2007) take an impact-oriented view of climate change. More recently, the IPCC framework considered vulnerability from a non-hazard perspective and separated the exposure component from vulnerability (IPCC 2014). All these frameworks have guided studies for framing and assessing vulnerability in different parts of the world.

Even though these frameworks have been widely cited in research, the range of application and contextual use of such frameworks have rarely been explored. Using an example of MOVE framework, the key objective of this paper is to assess the application of that framework in scientific discourse. In that, it help us whether or not assessment frameworks are practical in assessment of vulnerability, and what crucial aspects are needed to further improve framing of vulnerability given the development of the concept.

Therefore, this paper provides a systematic and thematic review of the MOVE framework, which was developed within the context of the European Commission FP7 research project – MOVE; [European Commission 2011]). The project aimed to improve methods for vulnerability assessment to natural and socio-natural hazards. This paper explores the application of the MOVE framework in assessing vulnerability in terms of hazard type, assessment approach, context and spatial scale. Moreover, in this paper, we also perform a bibliometric analysis of the MOVE framework publication and examined how the publication was cited in terms of publication titles and the field of study.

## Vulnerability assessment frameworks: A brief overview

Multiple views and institutional and academic perspectives on vulnerability have led to multiple conceptual and theoretical models and frameworks for assessment (Jamshed et al. 2019; Jamshed et al. 2020b). These frameworks and models have been widely used in various fields of study, for example, livelihood security, development, DRR and climate change adaptation. The frameworks help in defining problems and developing appropriate indicators and assessment methodologies. Moreover, frameworks help to focus on the most relevant factors (Birkmann 2013).

Several frameworks have been developed depending on the assessment approach, conceptual understanding of vulnerability, dimensions and spatial scales.^1^ Some frameworks focus on geographic/site condition, exposure and hazard impacts or simply view vulnerability as proximity to hazard (biophysical vulnerability), while some focus on socio-economic aspects (social vulnerability) while others include both biophysical and socio-economic aspects (integrated assessment) (Cutter et al. 2008; see Table 1).

**TABLE 1:** An overview of the main conceptual frameworks, including their type of assessment approach, their understanding of vulnerability, at what spatial scale they are applied and the key critiques of the framework.

Name of framework	Assessment approach	Conceptualisation of vulnerability	Vulnerability dimensions	Spatial scale	Critique
**Hazard of place model (1996)**	Integrated	Combination of biophysical and social vulnerability	Social, Geographical	Local/ place	Fails to account for larger spatial context, recovery/adaptation and the root causes of antecedent social vulnerability
**Sustainable livelihood framework (1999)**	Socio-economic	Shocks, trends and seasonality can be influenced by transforming structures	Human, Social, Financial, Physical, Natural	Local/ place	Abstract in terms of transforming structures and access to resources counts only for positive outcomes, while the feedback process underestimates the role of livelihood outcomes in hazard context
**Holistic approach (2000)**	Integrated	Function of exposure, susceptibility/fragility and ability to cope/recover	Social, Economic, Physical	Local to national	The classification of vulnerability conditions into ‘soft’ and ‘hard’ risk is debatable and environmental dimensions are not given due attention
**Vulnerability in the context of socio-ecological perspective (2003a)**	Integrated	Function of exposure, sensitivity and resilience	Coupled human and environment	Local to global	The framework does not clearly differentiate between exposure and sensitivity and has a missing temporal dimension that shows the start and end point of vulnerability. It is unclear whether the difference between drivers and consequences in a feedback-loop system is useful and. how to access the cross-scale interactions.
**The pressure and release (PAR) model (2004)**	Socio-economic	Explained by three progressive levels: root causes dynamic pressure and unsafe conditions	Physical environment, Local Economic, Social relations, Public action and institutions†	Local to global	Inadequately address the coupled human-environmental systems associated with proximity to hazard. It is difficult to distinguish between the causal links of different dynamic pressures on unsafe conditions and the impact of root causes on dynamic pressures. The framework gave due importance on national and global levels while several unsafe conditions and dynamic pressures might also be determined by local situations.
**BBC framework (2006b)**	Integrated	Function of exposure, susceptibility and coping capacity	Social, Economic, Environmental	Local/ place	Organisation and institutional aspects are not clearly defined and suggested for analysis within the three thematic spheres. The application scale is not defined, and the framework focuses on coping capacities while adaptive capacities are neglected.
**Second generation vulnerability assessment framework (2006)**	Integrated	Function of exposure, sensitivity and adaptive capacity	Not specified	Local to global	The framework focuses heavily on biophysical vulnerability, which includes hazard characteristics or physical characteristics of climate change that lead to risk, rather than a vulnerability assessment
**Intergovernmental Panel on Climate Change vulnerability and risk framework (2014)**	Socio-economic	Consists of susceptibility and capacity to cope and adapt	Environment, Social, Economic‡	Local to global	Interactions among multiple drivers of climate change risk and how multiple risks compound or cascade is not clear

*Source*: Adapted from Jamshed et al. 2020b with additional information from Birkmann, J. (ed.), 2013, ‘Measuring vulnerability to promote disaster-resilient societies and to enhance adaptation: Conceptual frameworks and definitions’, in *Measuring vulnerability to natural hazards: Towards disaster resilient societies*, 2nd edn., pp. 9–79, United Nations University Press, Tokyo; Cutter, S.L., Barnes, L., Berry, M., Burton, C., Evans, E., Tate, E. et al., 2008, ‘A place-based model for understanding community resilience to natural disasters’, *Global Environmental Change* 18(4), 598–606 https://doi.org/10.1016/j.gloenvcha.2008.07.013

† , As specified in the section that deals with ‘Unsafe Conditions’.

‡ , As specified in IPCC SREX Report 2012.

It is evident from Table 1 that some frameworks include components of exposure and hazards in the assessments, while others consider vulnerability a more societal issue and independent from hazards. However, these frameworks have been criticised regarding scales (Cutter 1996; Turner et al. 2003), conceptual understanding of central factors like components and dimensions (Füssel & Klein 2006; Turner et al. 2003), feedback systems (Department for International Development [DFID] 1999) and understanding complexity of interactions (IPCC 2014).

Table 1 also shows that most past frameworks focussed on integrated approaches. Birkmann (2013) argues that some integrated approaches consider hazard characteristics, which led the assessments to be not just about vulnerability but risk, for example, the ‘second-generation vulnerability assessment framework’ such as that deployed by Füssel and Klein (2006), which involves magnitude, charterer and duration of hazard. Furthermore, these frameworks can in theory be operationalised at various scales, but how to accomplish an actual cross-scale analysis of vulnerability is not clear (e.g., Turner et al. 2003).

In short, all frameworks have pros and cons. Several frameworks paved the way for the development of a more comprehensive framework. Thus, for instance, the BBC framework led to the MOVE framework, and the MOVE framework guided the IPCC’s framing of risk and vulnerability.

## The Methods for the Improvement of Vulnerability Assessment in Europe framework: A brief overview

**FIGURE 1:**
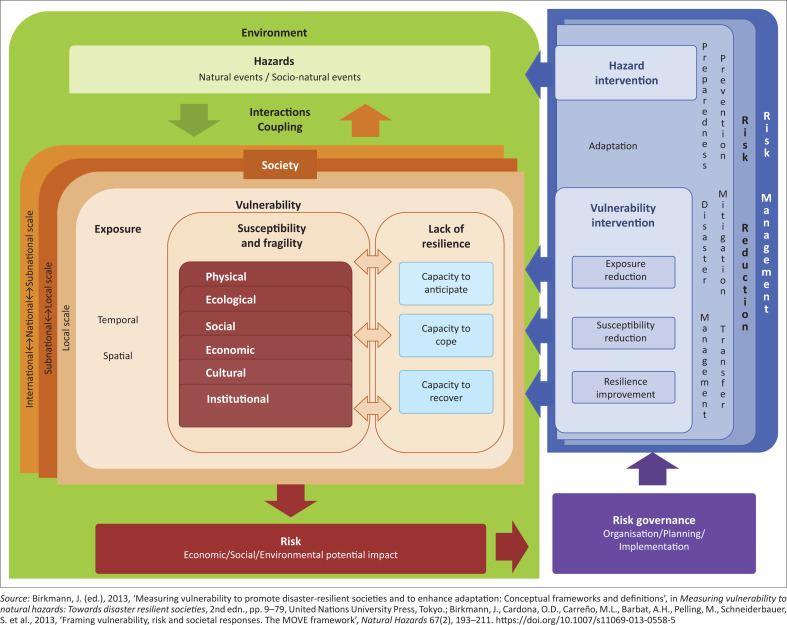
The Methods for the Improvement of Vulnerability Assessment in Europe framework.

Methods for the Improvement of Vulnerability Assessment in Europe is a framework for understanding multi-dimensional, holistic vulnerability in the context of disaster risk management and climate change adaptation. The conceptual framework is a pre-analytic vision that shows the linkages among key concepts, such as vulnerability, risk and adaptation. As a heuristic, MOVE is a thinking tool to guide systematic assessments of vulnerability and to provide a basis for comparative indicators and criteria developed to assess key factors and various dimensions of vulnerability (Birkmann et al. 2013). Consequently, the framework is a tool for communicating complexity and is not intended to serve as a detailed representation of processes and outcomes (Figure 1). The framework views society as embedded in the wider setting of the environment shaped by human actions (Birkmann 2013), which different than other frameworks developed in the past.

At the core of the framework are three key factors and six thematic dimensions. The key factors are: *exposure,* which is considered a hybrid between vulnerability and hazard, *susceptibility,* which shows the predisposition of elements at risk to suffer harm; and lack of *resilience,* which corresponds to deficiencies in societal response capacities. Thematic dimensions include the physical, ecological, social, economic, cultural and institutional dimensions. With the feedback loop systems, MOVE frameworks present multidimensional and process-oriented vulnerability and emphasise risk governance as a crucial element in the overall framing of vulnerability and risk (Birkmann 2013; Birkmann et al. 2013).

## Methodology employed to review uses of Methods for the Improvement of Vulnerability Assessment in Europe

This study uses two approaches. Firstly, a bibliometric analysis of the original research article in the journal *Natural Hazards* (Volume 67, 2013) was carried out to identify the type of documents, journals, research areas where MOVE was cited and in which countries. Doing this provided a comprehensive overview of how the original article has been utilised. To do so, we used the Web of Science database to search the title of the original paper. This provided us with the number of citations, research areas, document types, names of research scholars and countries, as well as keywords until date of analysis that is May 2022.

The results were extracted and graphs constructed using Microsoft Excel. VOSviewer 1.16.18 software was used to develop network maps and observe the co-occurrence of keywords (Figure 3). The font size of the keyword and the node/circle represent the weight of the keyword and links with other keywords. Thus, a larger node size shows that a particular keyword occurs with more keywords. The links between two keywords signify the linkages between them. Here, the thickness of the links represents the co-occurrence of both keywords together (Rana 2020; Wang, Zhao & Wang 2018). However, some keywords might not appear in the figure because of overlapping with other major keywords. The colour of the cluster determines the colour of the node to which the keyword belongs (Waltman, Van Eck & Noyons 2010).

Secondly, a qualitative approach to analyse the specific research documents that used the MOVE framework for vulnerability assessment. In Web of Science, Boolean operators with keywords ‘MOVE Framework AND vulnerability’ and ‘MOVE Framework AND vulnerability assessment’ were used to search the titles, abstracts and author keywords and, as a result, identify articles and/or documents that applied the framework. The database was searched on 02nd May, 2022. Using the retrieved search results, we developed criteria to check in which scientific discourse, at what spatial level, for what type of hazard and in which geographical area the framework was utilised. This provides an overview of the acceptability and usability of the MOVE framework paper.

## Results and discussion

### The use of the Methods for the Improvement of Vulnerability Assessment in Europe framework in research

The original article MOVE has been cited 462 times according to the Web of Science database as of 02nd May 2022. We took the Web of Science database as it contains only peer-reviewed material. The data in Web of Science are disaggregated enough to perform a detailed bibliometric analysis (Rana 2020).

**FIGURE 2:**
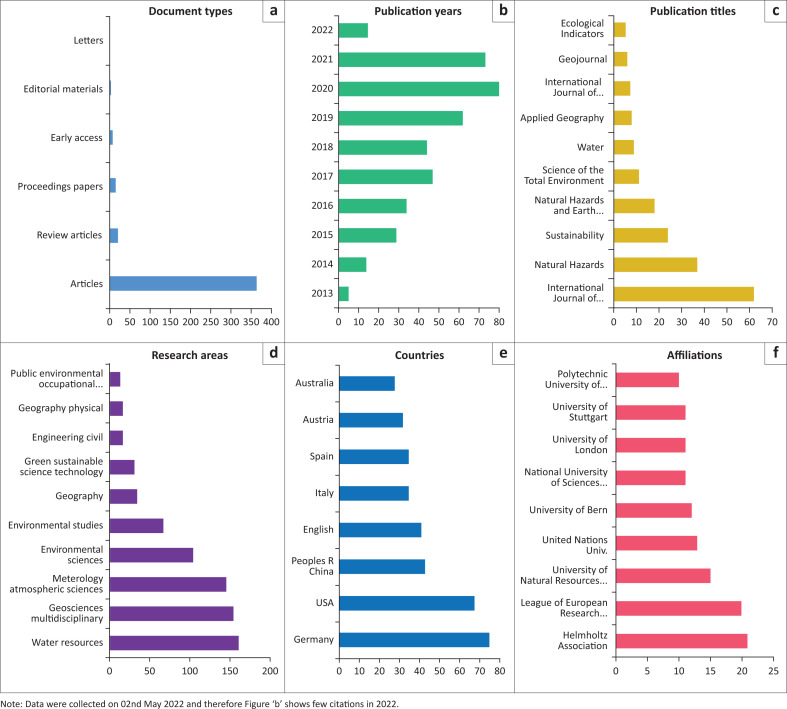
(a) Type of documents citing MOVE; (b) Annual citations of MOVE; (c) Top 10 journals citing MOVE (out of 166 journals); (d) Top 10 research areas citing MOVE (out of 68); (e) Top 10 Countries citing MOVE (out of 84 countries); (f) Top 10 affiliations of others citing MOVE (out of 695).

The annual publications show a steady increase in the citations of the MOVE framework, implying a growing interest in academia. In terms of document types, research articles were the main document type (more than 80%). This shows that the paper was predominantly cited in original research studies and some review articles (Figure 2a).

In terms of the number of annual citations of the paper, it was found that citations of the MOVE framework paper have increased every year since its publication (see Figure 2b). The article was highly cited in 2020 and 2021, with 80 and 72 citations, respectively. Only the year 2018 and 2021 has slightly fewer citations compared to the previous year, but overall a clear linear increase can be seen. This shows that scholars are increasingly using the information in the paper to support their research work.

Furthermore, we found that there were 365 publication titles (journals) in which the article was cited (see Figure 2c). The *International Journal of Disaster Risk Reduction* published the highest number of articles that cited the MOVE framework, followed by *Natural Hazards*. Looking only at the top 10 journals, it is evident that the MOVE framework has been cited in hazard or disaster risk-related journals and other journals that deal with broader topics of environment, sustainability and geography.

Regarding the research areas, our analysis shows that there are 68 research areas (according to Web of Science categories) that cited the MOVE framework paper. This indicates that the paper has been cited in diverse and multidisciplinary research areas that deal with hazard vulnerability in a social, natural and physical science context. The main research areas citing the article were water resources, geosciences, meteorology, atmospheric sciences, environmental sciences and sustainability science (see Figure 2d).

We also looked at countries citing the MOVE framework paper in their research. Germany, the United States of America (US), China, England and Italy were the top five countries citing information from the MOVE framework paper. Scholars in Germany and the US cited the article the most, with 75 and 68 citations, respectively (see Figure 2e). Overall, apart from China, all the top 10 countries were from the Global North. In terms of institutions to which citing researchers were affiliated, Helhomlz Institution cited the MOVE publication the most (see Figure 2f). It was also noted that the majority citing the paper were affiliated with European institutes.

The top-10 authors that cited the frameworks were from the research fields of DRR, climate change adaptation and sustainable development. Steven Fuchs, Irfan Ahmad Rana and Micheal Hagenlocher were the top-three researchers emphasising the importance of the MOVE framework in reducing disaster risks and mitigating climate change impacts.

#### Analysis of keywords

Keywords are chosen purposefully by the author(s) to increase the searchability of a publication. The keyword analysis reveals insights into similar concepts, ideas, approaches or debates. The keyword analysis showed the top-10 keywords used by the authors that cited the MOVE framework. These included vulnerability, resilience, climate change, risk, adaptation, exposure, social vulnerability, climate change adaptation, risk assessment and agriculture.

**FIGURE 3:**
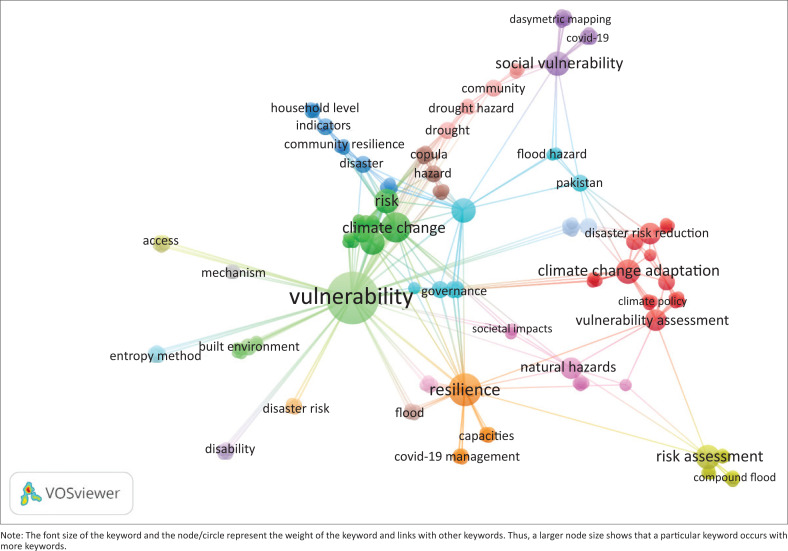
All authors’ keywords (203 keywords). VOSviewer 1.16.18 software to develop network maps and observe the co-occurrence of keywords. The font size of the keyword and the node/circle represent the weight of the keyword and links with other keywords.

The retrieved results revealed 203 distinct keywords (Figure 3). The Figure represents all keywords used by authors. The top-five keywords used were vulnerability, resilience, climate change, risk and adaptation. These keywords are extensively utilised in DRR and climate change adaptation discourse (Janssen et al. 2006; Rana 2020). Many scholars have emphasised the linkages among the concepts of vulnerability, resilience and adaptation (Adger 2006; Folke et al. 2010; Janssen & Ostrom 2006; Sapountzaki 2012). Other bibliometric studies (see e.g., Janssen et al. 2006; Kim, Jeong & Chung 2021; Rana 2020; Rufat et al. 2015) of climate change and DRR literature have also found vulnerability to the most frequently used word (Rana 2020; Wang, Zhao & Wang 2018).

#### Analysis of published papers using the Methods for the Improvement of Vulnerability Assessment in Europe framework

The MOVE framework has been used to assess vulnerability in case studies. This section examine selected studies on how the MOVE framework was applied, for example, in terms of factors or dimensions, location of case study, type of assessment, type of hazard and spatial context (see Table 2).

**TABLE 2:** Selected studies utilising the Methods for the Improvement of Vulnerability Assessment in Europe framework for vulnerability assessment or used it as a basis for developing a study-specific framework.

Research studies	Scientific discourse	Factors/dimensions	Case study	Type of assessment	Type of hazard	Spatial context
Rudolph-Cleff et al. (2022)	Disaster risk	Factors	Darmstadt, Germany	Quantitative	Power failure	Local (Urban)
Mason et al. (2021)	Disaster risk and climate change	Factors	Aotearoa New Zealand	Quantitative	Flooding	Local (Urban)
Hamidi et al. (2020)	Disaster risk	Factors	Peshawar, Pakistan	Quantitative	Flooding	Local (Urban)
Gomez et al. (2020)	Climate change	Factors	Gunjur, Gambia	Quantitative	Coastal Erosion	Local (Rural)
Leis and Kienberger	Climate change	Factors	Austria	Quantitative	Flooding	Sub-national
Leis and Kienberger (2020)	Disaster risk and climate change	Factors	Tokyo, Japan	Quantitative	Flooding	Local (Urban)
Dongo, Kablan and Kouamé et al. (2018)	Climate change	Factors	Abidjan, Cote d’Ivoire	Quantitative	Heat	Local (Urban)
Jackson, McNamara and Witt et al. (2017)	Disaster risk	Dimensions	Emae Island, Vanuatu	Qualitative	Multi-hazard	Local (Rural)
Kablan, Dongo and Coulibaly (2017)	Disaster Risk	Factors	Cocody, Cote d’Ivoire	Quantitative	Flood	Local (Urban)
Welle et al. (2015)	Disaster Risk	Factors	Cologne, Germany	Quantitative	Multi-hazard	Local (Urban)
Sané et al. (2015)	Disaster risk	Factors	Medina Gounass, Senegal	Quantitative	Flood	Local (Rural)
Tedim et al. (2015)	Climate change	Factors and dimensions	Portugal	Quantitative	Forest Fire, Coastal Erosion	Municipal (with urban and rural)
Bizimana, Twarabamenye and Kienberger (2015)	Disaster risk	Factors (without exposure)	Rwanda	Quantitative	Human health (Malaria)	Sub-national (district)
Leis and Kienberger (2014)	Disaster risk	Factors (without exposure)	East Africa	Quantitative	Human health (Malaria)	Sub regional, National and Sub-national
Depietri, Welle and Renaud (2013)	Climate change	Factors	Cologne, Germany	Both quantitative and qualitative	Heat waves	Local (Urban)
Hagenlocher et al. (2013)	Disaster risk	Factors (without exposure)	Cali, Columbia	Both quantitative and qualitative	Human health (Dengue)	Local (Urban)

Methods for the Improvement of Vulnerability Assessment in Europe framework offers factors (exposure, susceptibility, lack of resilience) and dimensions (social, economic, physical, institutional, etc.) as tools to assess vulnerability. The analysis of selected studies suggested that some focussed on dimensions; while others emphasised factors. Use of factors was found to be more common because factors offer an easy and broad spectrum for selecting indicators. Moreover, these factors have been significantly discussed and debated in past research studies (e.g., Birkmann 2006; Füssel 2005; Hamidi et al. 2020; Mason et al. 2021 Rudolph-Cleff et al. 2022; Turner et al. 2003 etc.). Methods for the Improvement of Vulnerability Assessment in Europe’s dimensions, by contrast, offer an understanding of different types of susceptibilities and vulnerabilities. Tedim et al. (2015) used multiple dimensions and identified indicators for each dimension.

Some studies using the MOVE framework exclude the exposure dimension (Bizimana et al. 2015; Hagenlocher et al. 2013). These studies consider vulnerability to be a predisposition of a population or system to be adversely affected by a hazard event. This predisposition is characterised by susceptibility and lack of capacities or resilience. The MOVE framework considers exposure as a hybrid concept between vulnerability and hazards (Birkmann et al. 2013). General exposure of a location can be a part of the hazard, but the degree to which a systems’ elements fall in hazard-prone areas depends on spatial and temporal dimensions of exposure and can thus be a part of vulnerability assessment.

The hazards considered by MOVE may be natural, socio-natural and anthropogenic hazards. Our analysis suggested that studies have applied the MOVE framework to assess vulnerability to all these kinds of hazards. Most of the research studies applied MOVE in the context of flooding (e.g., Hamidi et al. 2020; Kablan et al. 2017; Lianxiao & Morimoto 2019; Mason et al. 2021). Indeed we saw above that a large proportion of studies citing MOVE deal with water resources (see Figure 2d). In addition, the framework has been applied (see Table 2) to individual climatic hazards (e.g., heat, forest fires, coastal erosion) and to multiple-hazards (e.g., combination of floods, droughts, earthquake), as well as anthropogenic hazards such as power failure, and to socio-natural hazards such as the diseases of dengue and malaria.

The MOVE framework guided these studies to select hazard-dependent and independent indicators that represent the susceptibilities and capacities of populations and entities. For example, Hamidi et al. (2020) utilised hazard-dependent indicators like injuries from the flood and early warning of a past flood event. Kablan et al. (2017) used indicators such as method of water collection and unplanned waste disposal that can lead to clogged drainage systems and increase flood occurrence. Bizimana et al. (2015) used, among others, more health-related indicators (number of health facilities, nurse-to-population ratio, malnutrition, etc.) to assess vulnerability to malaria. Leis and Hagenlocher (2014) used indicators like distance to hospital, HIV prevalence, immunity, among others to assess social vulnerability to malaria in East Africa.

The MOVE framework does not provide specific qualitative or quantitative assessment methods. The analysis of selected case studies suggests MOVE has been applied for both qualitative and quantitative vulnerability assessment. The majority of studies used it for quantitative assessments using different analytical approaches. Index-based assessment was the key analytical approach for quantitative vulnerability assessment. Some studies used multivariate analyses such as principal component analysis (Bizimana et al. 2015; Hagenlocher et al. 2013) to group indicators, to check their robustness. Studies have used different aggregation equations for quantifying vulnerability (Bizimana et al. 2015; Hamidi et al. 2020). Depending on the scale of the study, the results of the assessment were visualised in the form of geographic information system (GIS)-based maps, graphs and tables.

Some studies have used the MOVE framework for qualitative vulnerability assessment based on stakeholder interviews, informal discussion, transect walks and participant observations to identify the causal factors of vulnerability and its components (Jackson et al. 2017). The MOVE framework guided the major themes, for example, social, economic, cultural, among others, along which these investigations were done.

In terms of spatial context, the MOVE framework recognises characteristics that define vulnerability as typical or valid at a certain scale. It also considers that a particular scale corresponds with the different needs of people or institutions at different times. The framework has been applied at various spatial levels, from local to national. However, most of the studies were conducted locally, assessing the vulnerability of rural or urban municipalities and households.

The MOVE framework has been used to guide case studies in different parts of the world, including Europe, Latin America, Asia, Africa and Oceania. In each case study, the MOVE framework guided indicator development that is context and hazard specific for assessing vulnerability. Our analysis suggested that the MOVE framework – even though it was developed in the context of improving vulnerability assessment in Europe – has been a valuable tool for vulnerability analysis in other parts of the world at various spatial levels.

#### Conceptual developments based on the Methods for the Improvement of Vulnerability Assessment in Europe framing of vulnerability

Several studies have used the MOVE framework to develop further context-specific frameworks (see Table 3). For example, Hamidi et al. (2022) developed a framework to assess vulnerability to flooding in rural areas of Pakistan, considering three components of MOVE. Ramli et al. (2021) developed an integrated disaster risk and vulnerability assessment framework for Malaysia based on the dimension of the MOVE framework.

**TABLE 3:** Selected studies that used the Methods for the Improvement of Vulnerability Assessment in Europe framework to develop a study-specific or extended framework.

Research studies	Scientific discourse	Factors/ dimensions	Case study	Type of hazard	Spatial context
**Hamidi et al. (2022)** for vulnerability assessment	Disaster risk	Factors	Charsadda, Pakistan	Flooding	Local (Rural)
**Ramli et al. (2021)** for integrated risk and vulnerability assessment	Disaster risk	Dimensions	Malaysia	Multi-hazard	Multiple spatial scales (state, district, municipal and mukim)
**Jamshed et al. (2020b)** for vulnerability assessment based dynamics of rural-urban linkages because of flooding	Disaster risk and climate change	Factors and dimensions	Pakistan	Flooding	Local (both rural and urban)
**Kloos et al. (2015)** for multi-hazard risk and vulnerability assessment	Disaster risk and climate change	Factors and dimensions	West African Sudanian Savanna zones	Multi-hazard	Multiple spatial scales
**IPCC (2014)** for risk and vulnerability assessment natural and climatic hazards	Disaster risk and climate change	Factors	NA	Multiple natural and climatic hazards	Multiple spatial scales

Jamshed et al. (2020a, 2020b, 2020c) developed a framework to assess vulnerability considering the dynamics of rural-urban linkages because of flood hazards using different thematic dimensions and components from the MOVE framework (Jamshed et al. 2020b). This framework stresses spatial dimensions, for example, the role of proximity and size of cities for local rural vulnerability assessments (Jamshed et al. 2020a, 2020c).

Kloos et al. (2015) developed a framework based on the MOVE to assess multi-hazard risk and vulnerability, explicitly focussing on the potential impacts of single and multiple hazards affecting socio-ecological systems. Their approach sought to be more flexible in linking resilience and vulnerability in a common framework for assessment of risk assessment. In the words of Kloos et al. (2015:26), ‘[our approach] accounts for societal response mechanisms through coping, adaptation, disaster risk reduction, and development activities which may foster transformation or persistence of the social-ecological systems’. Although the framework was developed for a specific spatial context for example, West Sudanian Savanna Zone, we find that the links between different elements is a bit complex and feedback loop system is missing. Moreover, it is unclear how the transformation would influence future risks and vulnerabilities.

The development of the MOVE framework also influenced the IPCC risk framework found in the SREX report (IPCC 2012). Intergovernmental Panel on Climate Change insists that it is essential to understand vulnerability within a broader system framework, which means differentiating a hazard that influences exposure and interacts with vulnerability. In this regard, the IPCC SREX framework takes up important aspects of the discourse about a holistic framework for understanding vulnerability (Cardona 2001; Cardona & Barbat 2000) as well as a system-theory based framework (i.e., based on systems thinking and considering feedback-loops) that is also implied by the MOVE framework (Birkmann et al. 2013). While MOVE gives more emphasis to different thematic dimensions of vulnerability, it is evident that both the IPCC SREX framework and the MOVE framework follow the same logic – an external stressor or hazard influences exposure and human vulnerability, and the outcome is a risk; however, risk and its determinants are not static but are influenced by present and future capacities of societies to influence the vulnerability of the exposed system and the hazard sphere (Birkmann et al. 2013; IPCC 2012).

**FIGURE 4:**
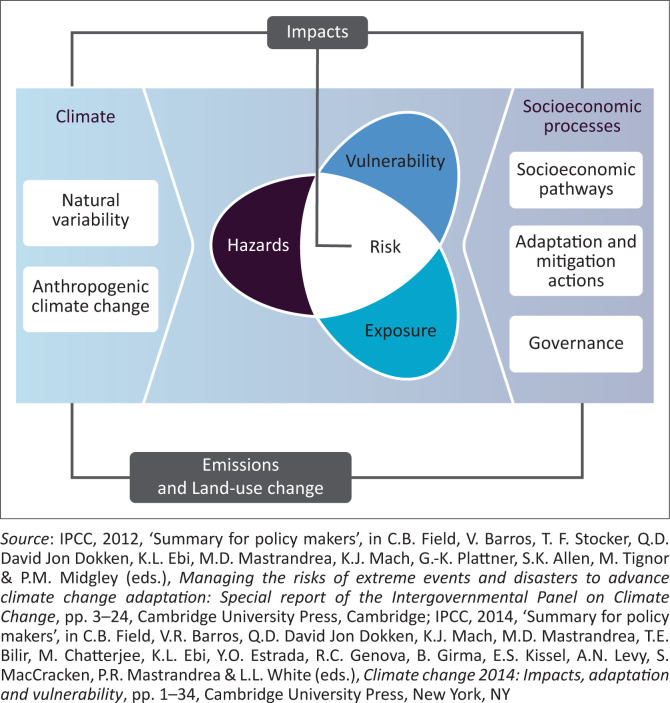
Intergovernmental Panel on Climate Change risk and vulnerability framing.

In the MOVE Framework, conceptualisation is strongly influenced by thinking about risk governance; while the IPCC framework was additionally informed by the climate change community, and therefore major factors that modify climatic hazards are included such as natural climate variability and anthropogenic climate change. A figure emerged in an IPCC meeting (Figure 4) that was created by the IPCC technical support unit and scientists (particularly Kristie Ebi) that bridges the climate and risk communities.

A key benefit of the new IPCC framework is the fact that a pure climate impact-driven understanding of climate change was modified towards a better understanding of core determinants of risk, including societal development pathways that influence exposure and human vulnerability. Also, the feedback-loop system provides a more dynamic understanding of how risks emerge in the context of climatic and societal change (Birkmann 2013; IPCC 2012, 2014).

Both the MOVE and IPCC frameworks underscore vulnerability and exposure as influenced by governance mechanisms and socio-economic development trends. The feedback loop system in these frameworks argues that people influence climatic or natural hazards, as well as exposure and vulnerabilities. Risk levels and potential and actual impacts shape risk governance, which in turn alters risk determinants of hazards.

### Critiques of the Methods for the Improvement of Vulnerability Assessment in Europe framework

As discussed, the MOVE framework was well accepted for the assessment of natural, socio-natural and human-induced hazards. At the same time, some critiques were made on the framework. Kloos et al. (2015) argued that in the MOVE framework society and social systems are the centres of analysis rather than socio-ecological systems. They positioned interaction between the environment and society as belonging conceptually to the domain of hazards. Further, they took issue with resilience being part of vulnerability in the MOVE framework, as they pointed out that several authors had argued that vulnerability and resilience are linked and, to some extent, overlapping and that integrating resilience into vulnerability might be problematic (Cutter et al. 2008). Thus, a more flexible approach is needed to link vulnerability and resilience (Kloos et al. 2015).

Jamshed et al. (2020b, 2021) also underscored the importance of interaction between spatial units (e.g., linkages between rural and urban areas) and criticised MOVE and other frameworks that do not clearly indicate the interaction between spatial units (Jamshed et al. 2020b, 2021). They argued that linkages between spatial units and especially their dynamics because of hazardous events are key in influencing vulnerability, which has rarely been considered in past vulnerability assessment frameworks like MOVE (Jamshed et al. 2020, 2021).

The MOVE framework comprehensively covers the topics of disaster risk management and climate change adaptation. However, disaster and climate change science are continuously evolving. The framework lacks the resilience concept’s dynamic nature and translates it from a capacity point of view. Similarly, the increasing importance of climate change mitigation is missing in the framework. The framework could also benefit from considering multi-hazard and complex/cascading risks. The attitudinal and psychological aspect of how individuals and communities react is limited in the framework. Similarly, compound drivers of risk and adaptation could have been highlighted. Although spatio-temporal scales are covered in the framework, it lacks the dynamic and uncertain nature of disaster and climate risks.

## Conclusions

The MOVE framework proved to be a holistic framework that provided the basis for vulnerability assessment to different hazard types. It acted as an important tool to develop indicators specific to natural or socio-natural hazards and the geographical context. It helped to identify indicators of different thematic dimensions. The power of the framework is to assess vulnerability to natural and climatic hazards, but analysis of the literature suggests that the framework has also guided the assessment of technological and socio-natural hazards like power outages and malaria. The literature review suggests that the MOVE framework received a good reception and high visibility in the various scientific communities and different parts of the world.

The framework shows its potential to guide the development of further frameworks by integrating concepts from other domains such as rural and urban development. The influence of the MOVE framework and its link with the IPCC framework was crucial to vulnerability and risk assessment around the globe (Roy et al. 2021; Sharma & Ravindranath 2019) and has provided a more standardised understanding of different concepts, for example, hazard, exposure, susceptibility, coping and adaptive capacity.

Overall, our literature review helped us understand the reception of the MOVE framework and its use in developing vulnerability assessment methodologies in different parts of the world. Critique of the framework can be helpful in further improvement and development of a multi-hazard holistic framework that would be flexible enough to support multiple theoretical perspectives in disaster risk and climate change discourses.

## Footnotes

1 For example, Birkmann (2006), Birkmann et al. (2013), Cardona and Barbat (2000), Cutter (1996); DFID (1999); Füssel (2005); IPCC (2014); Turner et al. (2003); Wisner et al. (2004).
